# The Infant Oral Microbiome: Developmental Dynamics, Modulating Factors, and Implications for Oral and Systemic Health

**DOI:** 10.3390/ijms26167983

**Published:** 2025-08-19

**Authors:** Paula Olate, Ailín Martínez, Eulàlia Sans-Serramitjana, Matías Cortés, Rommy Díaz, Genisley Hernández, Erwin A. Paz, Néstor Sepúlveda, John Quiñones

**Affiliations:** 1 Center for Innovation and Technology in Meat Quality (CTI-Carne), Universidad de La Frontera, Av. Francisco Salazar 01145, Temuco 4811230, Chile; p.olate02@ufromail.cl (P.O.); m.cortes10@ufromail.cl (M.C.); rommy.diaz@ufrontera.cl (R.D.); nestor.sepulveda@ufrontera.cl (N.S.); 2 Doctoral Program in Science Major in Applied Cellular and Molecular Biology, Universidad de La Frontera, Av. Francisco Salazar 01145, Temuco 4811230, Chile; a.martinez26@ufromail.cl; 3 Department of Integral Adultos, Facultad de Odontología, Universidad de La Frontera, Temuco 4780000, Chile; eulalia.sans@ufrontera.cl; 4 Center of Excellence in Translational Medicine—Scientific and Technology Bioresources Nucleus (CEMT—BIOREN), Universidad de La Frontera, Temuco 4811230, Chile; 5 Faculty of Agricultural and Environmental Sciences, Universidad de La Frontera, Av. Francisco Salazar 01145, Temuco 4811230, Chile; genisleyh@gmail.com; 6 UWA Institute of Agriculture, The University of Western Australia, Perth 6009, Australia; erwin.pazmunoz@uwa.edu.au

**Keywords:** infant development, nutrition, oral dysbiosis, oral health, oral microbiome

## Abstract

The infant oral microbiome is a complex and dynamic microbial community that undergoes various transformations during human development. From birth, these microorganisms are modulated by factors such as birth type, nutrition, oral hygiene, hormonal changes, and environmental and socioeconomic conditions. These elements interact continuously, shaping the diversity and stability of the oral microbiome and consequently influencing the oral and general health of individuals. The main objective of this study was to review the literature on the evolution of the oral microbiome at different stages of growth, with special emphasis on the maintenance of dental homeostasis and prevention of pathologies such as caries and periodontitis. A bibliographic review of scientific databases was conducted, focusing on the last decade. In general, oral microbiome dysbiosis increases the risk of oral diseases and systemic conditions. Diet, parental practices, and horizontal transmission of bacteria from caregivers have been shown to modulate and influence the composition and functioning of the infant oral microbiome. Despite these advances, gaps remain in our understanding of the impact of the pediatric oral microbiome on long-term comprehensive health. Therefore, longitudinal research is needed to understand the development of the oral microbiome and its potential role in early prediction, prevention, and treatment of oral and systemic diseases.

## 1. Introduction

The microbiome is defined as a characteristic microbial community that occupies a reasonably well-defined habitat and possesses distinct physicochemical properties [1]. The microbiome does not solely refer to the microorganisms involved, but also encompasses their functions, which lead to the formation of specific ecological niches [2]. Microorganisms colonize various areas of the body, including the skin, mucosa, gastrointestinal tract, respiratory tract, urogenital tract, and mammary glands [3,4]. These microbial ecosystems are composed of bacteria, archaea, fungi, and viruses that, to a large extent, live in a symbiotic relationship with their host [5]. There is no single human microbiome, but rather a wide range of possible configurations that reflect the coevolution between microorganisms and their hosts and play a fundamental role in health and disease [6]. These microbial communities perform diverse functions, regulating the immune system, producing bioactive metabolites, and protecting against pathogenic microorganisms, maintaining an adequate microbial balance in the oral cavity to prevent various systemic diseases [7].

In the last decade, interactions between the domains that compose the microbial community have been studied. These interactions are fundamental to the balance of the microbial ecosystem and can influence antibiotic resistance, virulence, and host immune response, particularly in viruses such as bacteriophages [8]. The oral cavity is the second largest and most diverse microbial community in humans, after the gut microbiota [9]. Data from the Human Oral Microbiome Database records approximately 774 microbial species, of which 58% are identified, 16% are culturable but unnamed, and 26% are unculturable phyla [10]. The phyla Bacillota, Bacteroidota, Pseudomonadota, Actinomycetota, Spirochaetota, and Fusobacteriota constitute 96% of the total. A healthy oral microbiome protects against pathogenic microorganisms, initiates food digestion, and regulates the immune system [11]. Dysbiosis, or an imbalance in the composition of the oral microbiome, can activate the inflammatory immune response of the host, causing oral diseases such as periodontitis, gingivitis, tooth loss, and oral cancer [12].

Oral diseases are the most prevalent health conditions globally and constitute a significant public health concern worldwide [13]. The most frequent cases are dental caries, periodontal disease, oral cancers, and tooth loss [14]. Notably, the WHO [15] reported that oral diseases affect an estimated 3.5 billion people worldwide, representing 45% of the global population, with a disproportionate burden of approximately three out of four cases concentrated in low- and middle-income countries [15]. Factors such as diet, antibiotic use, and poor oral hygiene may contribute to this microbial imbalance [16]. For these reasons, the adoption of proper oral hygiene practices is essential to avoid pathologies in the oral cavity. In addition, it should be kept in mind that the administration of antibiotics, oral antiseptics, probiotics, and prebiotics could influence the composition of the oral microbiome [7].

The oral microbiome develops dynamically from birth. The initial colonization dominated by species of the genus *Streptococcus* is influenced by multiple factors, such as the type of delivery, feeding, environment, and hormonal levels of the neonate. With the introduction of solid foods, a change in microbial composition is observed, with Bacillota and Bacteroidota predominating [17,18,19,20]. In this regard, early colonization may be altered by maternal and infant factors, binding site occupation, efficient nutrient use, antimicrobial agent production, and environmental changes [17,18,20]. Breast milk also plays an essential role in this process by transferring beneficial bacteria such as *Staphylococcus* spp., *Streptococcus* spp., *Bifidobacterium* spp., *Propionibacterium* spp., and *Lactobacillus* spp., whose composition varies according to the mother’s health status, genetics, antibiotic use, and dietary habits [21].

During early childhood, microbial diversity increases progressively, influenced by factors such as the type of delivery, contact with maternal microbiota, feeding, and physiological parameters of the host, including salivary cortisol levels, which significantly impact microbial composition [22]. Throughout childhood, the oral microbiome is organized in different niches, such as saliva, tongue dorsum, gums, and palate, where the genera *Streptococcus* and *Actinomyces* stand out as early colonizers and play key roles in dental biofilm formation [23].

An investigation indicated that neonates born via cesarean section present alterations in their microbiome compared to those born via vaginal delivery and that interventions such as vaginal microbiota transfer (VMT) can partially restore their microbial composition, accelerating their maturation and even showing positive effects on neurodevelopment at six months [24]. Similarly, breastfeeding is associated with a lower prevalence of *Streptococcus mutans* than formula feeding, suggesting a protective effect against dental caries. As children grow, the oral microbiome continues to transform in response to tooth eruption, dietary diversification, and progressive divergence from the maternal oral microbiota [25]. Beyond the oral environment, it has been suggested that the oral microbiome may maintain bidirectional communication with the central nervous system, opening new lines of research into its relationship with neuropsychiatric disorders and mental health [26].

Although significant advances have been made and it has been suggested that the oral microbiome could serve as a noninvasive biomarker to prevent oral and systemic diseases in childhood [27], there are still important gaps in knowledge about how the childhood oral microbiome relates to physical growth, neurodevelopment, and overall health [28].

In summary, the infant oral microbiome undergoes dynamic changes from birth to childhood, influenced by perinatal factors such as delivery mode and feeding type, with Streptococcus spp. being the earliest colonizers. During the transition to adolescence and adulthood, the microbial composition continues to shift in response to environmental exposures, diet, and lifestyle habits, such as tobacco or alcohol consumption. Oral homeostasis, which is closely linked to these compositional changes, may influence immune system development, the risk of oral diseases, and systemic conditions. However, longitudinal studies tracking these trajectories into adolescence are scarce, and the mechanisms connecting early life oral microbial patterns with long-term oral and systemic health remain poorly understood. Addressing these knowledge gaps is crucial for guiding preventive and therapeutic strategies from the earliest stages of life. Therefore, this review aimed to examine the literature on the evolution of the oral microbiome throughout the different stages of growth, with special emphasis on the maintenance of dental homeostasis and the prevention of pathologies, such as caries and periodontitis.

This review article was conducted using the scientific databases PubMedWeb, Web of Science, ScienceDirect, and Scielo, focusing on the literature published since 2000 and considering research from the last decade.

## 2. Definition and Composition of the Human Oral Microbiome

Bacteria emerged approximately 3.8 billion years ago and, along with archaea, protists, and fungi, have persisted for much of evolutionary history as individual free-living cells [29]. However, over time, some of these life forms began to establish associations with higher organisms, initiating coevolutionary processes that resulted in stable symbiotic relationships. Through coevolution, the microbiome has contributed significantly to shaping the phenotypes of ancestral lineages, influencing their biology and adaptation to the environment [29,30]. In this context, the term microbiome refers to the set of microorganisms, including bacteria, fungi, viruses, and archaea, as well as their genomes, that inhabit various niches in the human body [5]. In the last decade, the study of the human microbiome has gained increasing prominence, enabling a deeper understanding of both health and disease development. Among the different microbial communities, the gut microbiome has been the most extensively investigated. A relevant milestone in this field occurred in 2017, when experts from academia and industry participated in an annual symposium organized by the University Medical Center Groningen in the Netherlands. The meeting addressed the interactions between prebiotics, probiotics, and vitamins with the gut microbiome, highlighting their influence on multiple aspects of human health. These include modulation of the immune system, metabolism, and neurological functions, mainly mediated by the production of metabolites such as short-chain fatty acids [18].

The human oral cavity is the second most diverse microbial community, with more than 700 different bacterial taxa, while the first is the gastrointestinal cavity [31]. The anatomical diversity of the oral cavity configures different habitats, with different physical and chemical factors. These niches (lips, cheeks, palate, teeth, and gingival sulcus) are conducive to different microbial populations [32]. The aerial digestive tract (oral cavity, pharynx, nasal cavity, paranasal sinuses, and esophagus) harbors 775 bacterial species according to the Human Oral Microbiome Database (http://www.homd.org/, accessed on 17 July 2025). Among them, bacteria belonging to the genus *Streptococcus* are the most abundant in the mucosa; *Simonsiella* spp. are exclusive to the hard palate; and *Neisseria* spp., *Prevotella* spp., and *Haemophilus* spp. are abundant in other sites (tongue, saliva, and sub- and supragingival plaque) [33]. The Human Oral Microbiome Database (HOMD) provides comprehensive and selected information on bacteria present in the human mouth and aerodigestive tract, of which 58% are officially named, 16% are unnamed but cultured, and 26% are known only as uncultured phylotypes. The HOMD taxonomy describes oral bacterial taxa using a 16S rRNA identification tool and a repository of oral bacterial genome sequences [10]. In addition to bacteria, the oral cavity harbors fungi and viruses that significantly influence microbial ecology and oral health. Villar and Dongari-Bagtzoglou [34] highlighted that *Candida albicans* can proliferate in conditions of dysbiosis and low immune response, forming mixed biofilms with bacteria such as *Streptococcus*, which alters the composition of the microbiome and favors the onset of oral candidiasis in vulnerable hosts. In contrast, Xia and Pierson [35] show that human papillomavirus (HPV) infection is linked to changes in the oral microbiome that affect viral persistence, immune response, and the tumor microenvironment, further suggesting that the microbiome could serve as a biomarker and be modulated by probiotics in the context of prevention or treatment of HPV-related diseases. These findings broaden our understanding of the impact of non-bacterial organisms on the configuration of the oral microbiome and underscore the need for more comprehensive approaches to oral health prevention and diagnosis [34,35].

**Figure 1:**
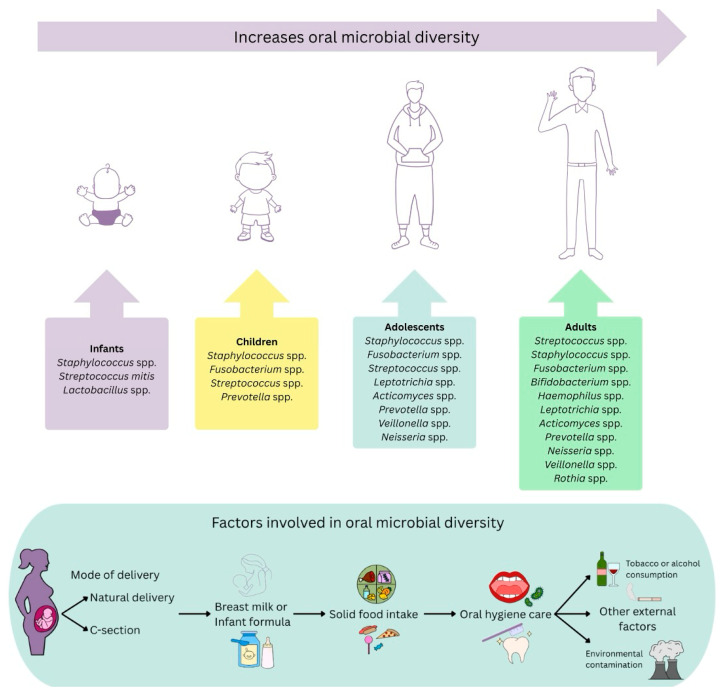
Evolution of the human oral microbiome. From the womb to adulthood: maternal and environmental aspects.

A characterization study of the oral microbiome in children and infants revealed the existence of a core microbiome that remains stable with the appearance of the first teeth, although its composition diversifies during the first four years of life (Figure 1). Bacteria of the phyla Pseudomonadota, Fusobacteriota, Actinomycetota, Bacteroidota, Bacillota, Synergistetes, Tenericutes, and bacteria of the genera *Neisseria* spp., *Kingella* spp., and *Leptotrichia* spp. were identified among the Operational Taxonomic Units (OTUs). In particular, the *Enterobacteriaceae* family becomes dominant between 12 and 24 months of age. In older children, *Streptococcus*, *Veillonella*, *Rothia*, *Prevotella*, *Fusobacterium*, *Actinobacillus*, *Neisseria*, and *Haemophilus* were predominant [36]. On the other hand, Xu et al. [28] compared the oral microbiome of children with and without caries, finding common genera such as *Streptococcus, Neisseria*, *Leptotrichia*, *Lautropia*, and *Haemophilus*. However, *Lactobacillus* spp., *Veillonella* spp., and *Prevotella* spp. were more abundant in children with caries, whereas *Neisseria* spp. were predominant in children without caries. In addition, *Veillonella* spp. have been shown to facilitate *Streptococcus mutans* adhesion and biofilm formation, thus contributing to the development of oral diseases such as caries and periodontitis [28,37]. This underscores the importance of understanding microbial dynamics during childhood to prevent oral pathologies.

### 2.1. Oral Microbial Colonization During the Lactation Stage

The origin of the oral microbiome in early life has been debated over the last decade. Some classical studies argue that the oral cavity of a newborn is sterile and that the intrauterine environment, including the amniotic cavity, is free of microorganisms [38,39]. However, more recent research has detected typical oral bacteria in amniotic fluid, suggesting possible prenatal colonization [40]. Furthermore, the existence of a uterine microbiome capable of contributing to fetal colonization has been proposed [41]. Nonetheless, current evidence on this phenomenon remains scarce and controversial; several authors have raised concerns that efforts to define prenatal microbiota may lack methodological robustness and conceptual clarity [42].

The current consensus is that microbial colonization of the oral cavity and gastrointestinal tract is initiated mainly after birth (Figure 1). Factors such as the type of delivery, breastfeeding, and environmental setting are fundamental at this stage, favoring the acquisition of exogenous and environmental bacteria [43,44].

Maternal health has become increasingly relevant in this context. It has been shown that the maternal oral microbiome can influence neonatal health, particularly when it harbors bacteria associated with periodontal diseases, such as *Porphyromonas* spp. and *Fusobacterium* spp., which have been linked to an increased risk of low birth weight [45]. During pregnancy, hormonal and immunological changes induce significant alterations in the composition of the oral microbiome, favoring the growth of species such as *Porphyromonas gingivalis*, which is related to elevated levels of progesterone and estrogen that favor its fumarate reductase system [46]. Fortunately, after childbirth, these modifications are usually reversed, restoring a more stable and healthier oral microbiota, largely due to the readjustment of sex hormones [47]. Furthermore, recent evidence suggests that maternal metabolic conditions, such as gestational diabetes mellitus (GDM), are associated with alterations in the composition of both maternal and neonatal gut microbiota. Although most research has focused on the gut microbiota, these microbial changes may also affect other mucosal environments, including the oral cavity [48]. This suggests that maternal metabolic disruption may indirectly predispose children to early oral microbial dysbiosis. These findings highlight the importance of considering maternal factors in assessing the development trajectory of the infant’s oral microbiota and its potential for long-term oral and systemic health [49].

### 2.2. Influence of Breastfeeding on the Establishment of the Oral Microbiome

Breast milk plays a fundamental role in the development of the neonatal microbiome (Figure 1). Khodayar-Pardo et al. [50] identified the presence of bacteria such as *Lactobacillus* spp., *Streptococcus* spp., *Enterococcus* spp., and *Bifidobacterium* spp. in human milk. Factors such as gestational age, mode of delivery, and lactation stage influence the bacterial composition of this milk. Although not all maternal bacteria are vertically transmitted, certain species can colonize and establish themselves within the infant’s oral microbiota. In addition, breast milk promotes the expression of protective immunological factors, such as antibodies, lactoferrin, beta defensins, and immune cells, which are transmitted to the infant in high concentrations [50,51,52,53,54].

Likewise, the mode of delivery influences the initial composition of the newborn’s oral microbiota. However, studies have also indicated that infants tend to share an oral microbiota similar to that of their mothers, suggesting possible hematogenous or intrauterine transmission [55]. With the eruption of the first teeth, microbial diversity increases due to the expansion of new niches in the oral cavity.

Reddel et al. [56] demonstrated that *Streptococcus* spp. and *Staphylococcus* spp. function as early commensals in the salivary microbiota of healthy neonates, whereas other genera such as *Fusobacterium*, *Prevotella*, *Porphyromonas*, *Granulicatella*, and *Veillonella* appear at later stages [56]. *Staphylococcus epidermidis*, a bacterial species commonly found on the maternal areolar skin, has been identified in both breast milk and the oral cavity of the newborn, suggesting a connection between the maternal skin and the infant’s oral microbiota [21,44].

Other studies have compared the oral microbiota of breastfed and formula-fed infants. Al-Shehri et al. [57], using 16S rRNA gene sequencing, found that formula-fed infants showed a higher abundance of Bacteroidota, whereas breastfed infants showed higher levels of Pseudomonadota [57]. In contrast, Butler et al. [58] identified that infants who were breastfed (for 10 months) showed a higher presence of beneficial genera such as *Streptococcus* and *Rothia*, while those who were never breastfed had a higher proportion of potentially pathogenic bacteria such as *Veillonella* spp. and species of the phylum Bacteroidota [58]. These findings support the hypothesis that breast milk is a key contributor to the development of a healthy oral microbiota during the first months of life (Table 1).

## 3. Development of the Oral Microbiome During Childhood

The oral microbiome undergoes continuous evolution, and the interaction between humans and their indigenous microbiota is becoming increasingly profound [3]. The first two years of life are crucial for the development of the oral microbiome, and the core oral microbiome increases to over 32 taxa at the species level [58]. The diversity and richness of the oral microbiome are known to be strictly related to taxonomy, including six major phyla: Bacillota, Bacteroidota, Pseudomonadota, Actinomycetota, and Spirochaetota [36]. Dashper et al. [62] investigated the evolution of the oral microbiome in 134 children from two months to four years of age and determined that the oral microbiome develops in a structured manner, with bacterial diversity increasing as the child grows (Table 2). The predominant bacteria identified included *Streptococcus mitis*, *Streptococcus salivarius*, *Rothia mucilaginosa*, *Neisseria subflava*, *Prevotella melaninogenica*, *Fusobacterium periodonticum*, and *Lautropia mirabilis*. In addition, from 19.7 months, some children exhibited the presence and abundance of *Streptococcus mutans*, which was significantly associated with the onset of early childhood caries [62].

The diversity of oral microbiota increases with the eruption of primary teeth, as demonstrated by Xu et al. [79], who found that even when infants had only two erupted lower primary central incisors, *Streptococcus* spp., *Rothia* spp., and *Haemophilus* spp. accounted for more than 70% of the total microbial abundance. These genera may represent the dominant taxa involved in the initial formation of supragingival plaque [79].

This finding is consistent with another study reporting that the primary dentition retains 85% of the core OTUs (Operational Taxonomic Units) identified before tooth eruption and that subsequent dentitions share over 90% similarity with the primary dentition in terms of phylogenetic and functional composition [80]. Moreover, microbial diversity and richness in infant saliva appear to increase with age, independent of maternal oral health status, educational level, mode of delivery, and breastfeeding practices [81]. Larger longitudinal studies have further confirmed that salivary species richness increases with age, with the most pronounced expansion in microbial diversity occurring between 3 and 18 months [82,83,84]. Historically, *S. mutans* and, to a lesser extent, *S. sobrinus*, have been implicated in the initiation of dental caries [85], particularly in children between 2 and 5 years of age—a critical period during which the oral bacterial community undergoes substantial development in the preschool stage [86]. Recently, other acidogenic and aciduric bacterial taxa, including various *Streptococcus* spp., *Actinomyces* spp., and *Bifidobacterium* spp., such as *Scardovia wiggsiae*, have also been associated with dysbiosis within the oral microbial community [87,88].

### Environmental Factors and Maternal Transmission in Infant Oral Health

Tao et al. [89] compared the oral microbiomes of mothers with and without dental caries and those of their 12-month-old infants using high-throughput sequencing. They found a positive correlation in oral microbial diversity between mothers and their infants, suggesting that maternal oral microbial communities influence the composition of the infants’ oral microbiome. Although *Neisseria*, *Streptococcus*, and *Kingella* were the most abundant genera in infants, no dental caries were observed [89]. These findings are consistent with other studies analyzing caries-free 3-year-old children, where the most frequent genera were *Kingella, Capnocytophaga*, and *Neisseria* [90], as well as another study that examined supragingival plaque, saliva, and tongue coating samples from healthy children aged 4 to 5 years, where bacteria associated with periodontal diseases were found, such as *Prevotella* spp., *Fusobacterium* spp., *Capnocytophaga* spp., and *Tannerella* spp., which are usually commensal species, but, under certain conditions, could form microbial complexes capable of inducing inflammation [23].

In another study, Li et al. [91] analyzed the oral microbiota of mothers and their infants and reported that *S. mutans* was detected in 96.8% of maternal saliva samples at the beginning of this study and in 100% of samples at follow-up visits. In contrast, *S. mutans* was not detected at birth, but it was present in 15.4% of cases at 9 and 15 months of age [91]. These findings suggest that behaviors such as sharing food, beverages, or utensils may increase the risk of maternal transmission of *S. mutans* to infants [92]. Despite being a preventable condition, dental caries remains one of the most prevalent diseases globally, disproportionately affecting vulnerable populations, particularly in Indigenous and low-income communities, where structural barriers limit access to healthcare and preventive services [93,94].

Among the factors influencing microbial dynamics are early feeding practices, free sugar intake, and oral hygiene behaviors. The latest findings have proven the presence of *S. mutans* and *S. sobrinus* in the oral cavity of children aged 3–5 years. The presence of both bacteria in the oral microbiota is associated with early childhood caries and changes in oral hygiene, which are, in turn, related to the increased consumption of sugary beverages and sweets [95,96,97,98].

Other investigations have highlighted the importance of oral hygiene in children with neurological impairments. Fregatto et al. [99].reported a higher prevalence of *S. mutans* in the oral cavity of this population, with its presence being associated with increased plaque accumulation and a higher prevalence of periodontal disease. In addition to compromising oral health, this may facilitate colonization by other pathogenic bacteria. Therefore, these studies highlight the importance of oral hygiene and the need for extra care in this population [99,100]. In a similar study, Duarte et al. [101] analyzed the bacterial composition, salivary pH, and flow rate in the oral cavity of children and young people with neurological impairments and oropharyngeal dysphagia. They found that patients fed via gastrostomy exhibited a significantly more alkaline salivary pH than those who were orally fed [101]. According to other studies, these findings can be attributed to the absence of food in the oral cavity. When the salivary pH exceeds 5.5, dental plaque becomes supersaturated, leading to mineral deposition. This occurs in alternatively fed patients, as their plaque is not exposed to fermentable carbohydrates [102]. Additionally, patients in both groups harbored *Porphyromonas gingivalis*, *Tannerella forsythia*, and *Treponema denticola*. These microorganisms play specific roles in the aetiopathology of periodontal infections and are prognostic factors for periodontitis [103,104,105,106]. Therefore, the authors suggest that introducing food into the oral cavity, even for the purpose of salivary stimulation, may help maintain salivary composition and pH, thereby contributing to the oral health of children and young adults with neurological impairments who are fed via alternative routes [101].

In contrast, Finlayson et al. [75] examined multiple factors influencing oral hygiene practices in young children. A key finding was that the behavior modeled by parents and older siblings contributed significantly to children’s oral hygiene habits, as young children tended to imitate these routines. Therefore, it is recommended to assess the need for support, education, and resources aimed at improving parents’ and caregivers’ tooth brushing techniques to promote healthy behaviors from early childhood [75]. In line with this, Duijster et al. [107] observed that when both parents fail to maintain and reinforce the child’s hygiene routine, this has a negative impact on the child’s oral hygiene behavior [107]. In addition, large-scale epidemiological data from Das Gupta et al. [108], who analyzed more than 266 000 school-aged children from 72 countries, revealed significant disparities in tooth brushing frequency, with twice-daily brushing rates varying widely by region; for example, as low as ~41% in the Eastern Mediterranean and as high as ~83% in the Americas. These findings highlight the global challenges of establishing oral hygiene habits and efficient educational interventions for children’s caregivers to improve oral hygiene habits in diverse populations [109].

Moreover, the pediatric oral microbiome is a multifactorial ecosystem whose composition is shaped by bacterial transmission within the nuclear family. Several studies have highlighted mother-to-child microbial transfer, with evidence showing that mothers and their children share a greater number of bacterial species than the child–father relationship [63,67,109]. Similarly, several early factors and events are crucial for shaping the oral microbiome during childhood. These include teething interventions, such as the use of specific gels, toys, and wipes, as well as oral hygiene habits, ranging from the type of cloth or brush (whether silicone bristle or plastic) and the frequency of brushing, to the age at which toothpaste is initiated. However, despite their relevance, these aspects remain largely unexplored, highlighting the need for further research to better understand their impact on the healthy development of the oral microbiome of children [110]. This knowledge is essential for designing preventive strategies and promoting practices that enhance oral health from the earliest stages of life.

## 4. Changes in the Oral Microbiome During the Adolescent Stage

Adolescence is a period of profound hormonal, physiological, and behavioral changes that also affect the microbial community of the oral cavity [65]. At this stage, which is still being explored, the oral microbiome shows variations in composition and diversity [66], modulated by factors such as puberty, oral hygiene, diet, and the use of orthodontic appliances [111,112]. These changes may increase susceptibility to diseases such as caries or gingivitis, and could have major implications for an individual’s systemic health [68]. Related to this, the current findings suggest that, during adolescence, the oral microbiota and immune response undergo adaptive development, which may influence susceptibility to periodontal and systemic diseases [113].

Studies have shown that the most abundant phyla in the healthy oral cavity of adolescents are Bacillota, Bacteroidota, Pseudomonadota, and Actinomycetota, while the most predominant genera are *Streptococcus*, *Prevotella*, and *Veillonella* [114]. Similarly, in other reports comparing the oral microbiome between adolescents and adults, the genera *Abiotrophia*, *Granulicatella*, *Actinomyces*, *Rhizobium*, *Burkholderia*, and *Ralstonia* were found to be abundant in the oral cavity of adolescents [115]. In summary, puberty is an important period for changes in the oral microbiome, influenced by hormonal changes; consequently, the oral microbiome of adolescents begins to resemble that of adults [116]. However, the dynamism of the oral microbiome continues to change due to other factors, such as habits, diet, and oral hygiene [32].

Exposure to metallic elements has become more frequent in recent times, with the general population coming into contact with them through water, food, and the environment [117]. Even essential metals may be toxic at certain concentrations [118]. Consistent with this, Davis et al. [119] associated oral health conditions, such as dental caries, with the presence of metals in saliva, even at low concentrations. Associations were found between high levels of arsenic, antimony, and mercury with significant decreases in the genera *Neisseria*, *Granulicatella*, and *Abiotrophia*, and significant increases in *Streptococcus* and *Prevotella* species, thus being associated with dental caries and tooth lesions [119]. Some biological samples are currently considered better biomarkers for determining metals in the body, such as whole blood for the detection of lead [120], serum for copper and zinc [78], and urine for the detection of arsenic, cadmium, chromium, or mercury [77]. However, based on the above findings, it can be deduced that saliva has potential as a minimally invasive biomarker for detecting metals, especially for studies focusing on oral health [119]. In addition to its potential for detecting environmental exposures, saliva has been proposed as a useful diagnostic fluid for assessing host immune responses and oxidative stress during oral interactions. Orzechowska-Wylegala et al. [120] identified elevated levels of pro-inflammatory cytokines and antioxidant enzymes in the saliva of children with dentofacial infections, suggesting that salivary biomarkers may reflect both microbial activity and host responses. These findings reinforce the relevance of saliva as a minimally invasive means of monitoring oral health, especially during periods of increased susceptibility, such as childhood and adolescence [120].

Among the various factors influencing the composition of the oral microbiome during adolescence, oral hygiene plays a significant role [121]. Frequent tooth brushing has been shown to have a more marked impact on the relative abundance of oral bacterial genera than flossing or fluoride supplementation. Similarly, alcohol consumption among adolescents appears to affect the oral microbiome more than smoking [66]. Social and environmental factors may also play an important role in the composition of the microbiome [122]. Sundström et al. [67] observed that young adult children who still lived with their parents shared a higher proportion of OTUs with them than older adult children who had been living separately for years. This suggests that prolonged cohabitation may influence oral microbial similarity between family members, even in adulthood [67].

In this regard, Eriksson et al. [68] investigated the composition of the oral microbiome in 17-year-old Swedish adolescents by classifying the samples according to the presence or absence of caries and found significant differences in bacterial composition. Species such as *Scardovia wiggsiae*, *S. mutans*, and *Bifidobacterium longum* were associated with the presence of caries, with the latter being a predictor of new carious lesions at the 19-year follow-up period. These findings underscore the importance of maintaining good oral hygiene [68].

On the other hand, the diversity and composition of the oral microbiome evolve from adolescence to old age. This was reported by Stahringer et al. (2012), who found that in individuals aged 8–26 years, the abundance of the genus *Veillonella* decreased over time, whereas the genera *Actinomyces* and *Streptococcus* increased [69]. In line with these findings, Liu et al. [64] noted that among participants aged 11–65 years, within-sample diversity (alpha diversity) decreased as between-sample diversity (beta diversity) increased. Additionally, distinct bacterial genera have been identified across different oral sites, such as saliva, gingival crevicular fluid, and the dorsum of the tongue [64]. Other studies indicate that alpha diversity tends to be low from childhood through adolescence, peaks in young adulthood (between 20 and 40 years), declines again after age 40, and subsequently increases with age. After the age of 40, the relative abundance of the genus *Neisseria* decreases, whereas taxa such as *Streptococcus anginosus* and *Gemella sanguinis* increase [123,124]. Collectively, these findings emphasize that microbial succession varies by oral site, with adolescence and later adulthood representing key transitional phases in the core oral microbiome [64].

## 5. Interaction Between the Oral Microbiome, Oral Diseases, and Systemic Conditions

Oral health is an important component of a person’s overall well-being and quality of life. The FDI (FDI World Dental Federation) defines oral health as a fundamental component of physical and mental health and well-being. It reflects the physiological, social, and psychological attributes essential to quality of life, and is influenced by an individual’s changing experiences, perceptions, and expectations, as well as their ability to adapt to circumstances [125].

Oral diseases, although mostly preventable, represent a major challenge for health systems in many countries and affect a considerable proportion of the population. The World Health Organization (WHO) estimates that oral diseases affect nearly 3.7 billion individuals. Untreated tooth decay in permanent teeth is the most common health condition, affecting approximately 2.3 billion people, whereas approximately 530 million children suffer from tooth decay in primary teeth [14].

During tooth eruption, emerging teeth acquire a protective glycoprotein layer that triggers successive microbial colonization, leading to the development of complex communities of polymicrobial biofilms, called dental plaque [126].

Common diseases of the oral cavity, such as periodontitis and dental caries, are clear examples of how low alpha- and beta-diversity of the microbiome is associated with the disease state. However, in the current context of microbiome studies, it has not yet been conclusively established whether changes in microbial composition are a cause or a consequence of disease [127].

Dental caries affects people of all ages, from infancy to old age, and is the most prevalent non-communicable disease worldwide. More than one-third of the world’s population lives with untreated tooth decay [14]. Periodontal disease, characterized by chronic inflammation of the tissues supporting the teeth, is clinically identified by the presence of periodontal pockets larger than 6.0 mm, which is indicative of severe disease. This problem represents a major public health challenge, compounded by behavioral factors such as smoking and other risks associated with non-communicable diseases [128].

It has also been shown that oral diseases can influence the aggravation of systemic pathologies such as arterial hypertension, diabetes mellitus, osteoarthritis, cardiovascular diseases, and cerebrovascular diseases, especially in older adults, due to the persistent presence of pathogenic bacteria in the oral cavity [129]. Among other oral diseases, dental fluorosis is considered endemic in some countries, such as China and India [130,131]. This pathology results from excessive exposure to fluoride over prolonged periods and manifests as irreversible tooth discoloration and surface irregularities [76]. Recent studies have suggested that dental fluorosis may be related to an increased susceptibility to other oral diseases, such as caries and periodontitis [132]. Therefore, it is important to consider that available treatments for dental fluorosis, in addition to their aesthetic benefits, could contribute to the health of the oral microbiome and the prevention of oral pathologies [133].

On the other hand, other research has been conducted regarding the oral microbiome and some non-oral diseases. For example, Al-Sarraj et al. [134] analyzed the saliva of individuals with sickle cell anemia (SCA) and healthy individuals. Both groups showed a predominance of bacterial genera such as *Streptococcus*, *Fusobacterium*, *Prevotella,* and *Veillonella*, and common species such as *Rothia mucilaginosa*, *Prevotella scoposa*, and *Veillonella dispar*. However, healthy patients had a higher abundance of *Streptococcus salivarius*, *Actinomyces graevenitzii*, *Actinomyces odontolyticus*, and *Actinomyces georgiae*, suggesting that, although the overall composition of the microbiome is similar, there are variations in relative abundance that could influence the susceptibility of these patients to oral complications [134].

In line with this, another study found that in children with atopic dermatitis, there was a significant decrease in microbial diversity in the skin, oral cavity, and gut compared to healthy children. In addition, a reduction in metabolic pathways related to serotonergic synapses, arachidonic acid metabolism, and steroid biosynthesis was observed at all three sites. These findings suggest that atopic dermatitis in children is associated with reduced microbial diversity and functional alterations, both metabolic and genetic, in the microbiota of the skin, oral cavity, and gut [135]. Another similar study investigated changes in the oral and gut microbiota of obese and normal-weight children. It was observed that children with obesity had a significant alteration in microbial composition, with a higher proportion of bacteria of the phylum Bacillota compared to Bacteroidota, both in the oral cavity and the gut, reflecting a state of dysbiosis that could have a negative effect on the development of this condition. Furthermore, it has been proposed that the oral microbiome may modulate the gut microbiome composition through immunomodulatory mechanisms. These findings reinforce the need to consider the oral bacterial community as a component in the prevention of childhood obesity [61].

Although recent studies have shown that certain systemic diseases may be associated with reduced microbial diversity in the oral cavity [134,135,136,137], most evidence suggests the opposite: oral diseases or disruptions in the oral microbiome are contributing factors to the development and prevalence of systemic diseases [138,139,140,141]. Although further studies are needed to fully understand the role of the oral microbiome in systemic health, several authors agree that both oral dysbiosis and oral and systemic diseases are strongly linked [7,142,143].

## 6. Influence of Food on the Formation of the Infant Oral Microbiome

Since the 1960s, dietary styles have changed, characterized by the consumption of certain foods such as farmed meat, high-sugar dairy products, refined oils, and processed grains [144]. Current research has shown that a balanced diet, rich in nutrients and low in sugars and ultra-processed foods, supports microbial diversity (Table 1). Furthermore, it has been concluded that Western dietary habits, characterized by a high consumption of processed meats, sugar-rich dairy products, refined vegetable oils, and processed cereals, may alter the oral microbial composition, favoring an increase in acidogenic microorganisms and periodontal pathogens [74]. In contrast, the Mediterranean diet, which contains anti-inflammatory and antioxidant-rich foods, has a positive impact on oral health [145].

Feeding at weaning is associated with distinct changes in the microbial community structure, including the oral microbiome [146]. Among studies analyzing oral samples from infants, exclusive breastfeeding has been shown to coincide with lower bacterial diversity on the tongue and cheeks, characterized by a predominance of *Streptococcus* spp. and reduced levels of *Prevotella* spp. In contrast, infants who received formula along with solid foods exhibited greater oral diversity and increased levels of *Prevotella* spp. and Fusobacteriota [60]. Other studies, focusing on the gut microbiome, have reported that the introduction of complementary foods, such as cereals, fruits, vegetables, and even a Mediterranean diet, is associated with increased microbial diversity and alterations in key taxa and metabolic products. Therefore, similar associations may exist with the oral microbiome [59,147,148,149]. In a clinical trial, Bartha et al. [70] demonstrated that gingival inflammatory parameters were significantly reduced in 42 participants after a six-week intervention with a Mediterranean diet. In addition, improvements in body weight and waist circumference were observed, indicating that macronutrient intake may contribute to a reduced oral and systemic inflammatory state [70]. Moreover, in a study involving high-performance athletes, a ketogenic, low-carbohydrate, high-fat diet altered the abundance of key oral bacteria (*Haemophilus* spp., *Prevotella* spp., and *Neisseria* spp.) involved in nitric oxide production. This shift was accompanied by an increased relative abundance of *Streptococcus* spp., suggesting a potential reduction in exercise efficiency in athletes. These findings highlight the important implications of diet for both athletic performance and oral health [71].

Other research has provided evidence that a high abundance of Streptococci—particularly *Streptococcus mitis* (accounting for approximately 9.5% of all detected species)—along with a low carbohydrate intake, was associated with the maintenance of dental health. When *Streptococcus mutans* is present at high levels, its fermentation of simple carbohydrates into lactic acid can promote the growth of acid-tolerant bacteria, such as *Cryptobacterium* spp., which are implicated in the development of dental caries [33]. These findings demonstrate that diet not only regulates microbial diversity in the oral cavity but also directly influences oral health and disease. The importance of adequate macronutrient intake, low sugar intake, and a balanced diet is, thus, emphasized for maintaining a healthy oral ecosystem, and preventing diseases such as dental caries and periodontitis is highlighted [150].

Studies on sugar consumption have shown that adolescents with caries and low sugar intake exhibit a higher abundance of cariogenic microorganisms, including *Streptococcus mutans*, *Scardovia wiggsiae*, *Lactobacillus* spp., and *Candida albicans*, than those without caries and high sugar consumption. Additionally, the caries group with low sugar intake showed increased expression of genes involved in sugar uptake and carbohydrate fermentation metabolism, such as phosphotransferase systems (PTS) and acidogenic pathways, suggesting that susceptibility to caries depends not only on sugar consumption, but also on the composition and metabolic activity of the oral microbiome, particularly its ability to import and ferment dietary sugars into organic acids that lower pH and promote tooth enamel demineralization [72]. In another similar study, the effects of different dietary sugars on the interaction between bacteria and fungi in saliva were investigated. There was an increase in *Streptococcus mutans* and *Candida albicans*, characterized by a glucan-rich complex structure, and dense, highly acidic biofilms developed. In contrast, in the absence of sugars, the microorganisms remained mostly as single cells without forming integrated structures, and the biofilms were sparse or less structurally integrated. Therefore, the importance of maintaining a nutrient-rich diet for good oral health and thus avoiding the initiation of pathogenic biofilms associated with dental caries or other oral diseases is highlighted [73].

## 7. Future Perspectives and a Comprehensive Approach to Children’s Oral Health

The study of the pediatric oral microbiome continues to expand, with various emerging areas focused on promoting comprehensive oral health and systemic well-being from the earliest stages of life. One of the most promising approaches is the modulation of the oral microbiome using probiotics, prebiotics, and specific dietary changes [151]. These strategies aim to promote the colonization of beneficial bacteria, reduce the proliferation of pathogenic microorganisms, and restore oral homeostasis [152]. While experimental and observational data support the potential of these interventions, their effectiveness in pediatric populations may vary depending on adherence, age at introduction, and baseline composition of the oral microbiome. For example, interventions based on an anti-inflammatory diet rich in antioxidants and low in sugars, such as the Mediterranean diet, have been proposed as sustainable tools for preventing oral diseases, such as caries and periodontitis, while supporting a healthy microbial ecosystem [145]. However, research data confirming that its impact on the composition and function of the microbiome in children is limited.

Parental education stands out as another determining factor. It is essential to reinforce proper oral care practices from early childhood, such as early tooth brushing, proper use of toothpaste, limiting sugar consumption, and regular visits to the dentist from an early age [75]. Although widely recommended, the actual adoption of these behaviors is influenced by knowledge, cultural norms, and socioeconomic status. Promoting healthy eating habits not only benefits a child’s systemic health but also contributes to the balance of the oral microbiome [153]. In this context, public health policies should prioritize parental education; however, their success will depend on the integration of these recommendations into accessible and culturally sensitive programs.

Another area of growing interest is the vertical and horizontal transmission of oral microorganisms between parents and children, especially in situations involving close contact, sharing of utensils, and sharing food. Numerous reports have shown that maternal oral health and health care awareness are directly linked to the oral health of infants and young children, which may have long-term implications for a child’s oral health [154]. Therefore, preventive strategies should include the appropriate treatment of periodontal diseases in adult caregivers and the promotion of family environments that support the development of a healthy oral microbiota in children. However, further research is still needed to better understand the relative contributions of maternal and environmental microbial transmission.

Finally, in addressing childhood growth delays, the role of the oral microbiome as a potential mediator between nutrition, systemic health, and growth should not be underestimated. Active parental involvement is crucial, particularly in providing adequate nutrition and preventing infection. Nutrient absorption in children can be influenced by multiple factors, including the presence and composition of the oral microbiome [155]. Understanding these interactions could open new preventive and therapeutic avenues; however, current evidence is insufficient, and further mechanistic studies are warranted. 

## 8. Conclusions

Current evidence demonstrates that the acquisition and maturation of the oral microbiome exert a significant influence on oral health and systemic health, particularly during critical stages of growth and development [156]. Multiple studies have highlighted the importance of early life practices, such as breastfeeding, a balanced diet, and adequate oral hygiene, in fostering microbial homeostasis, whereas unfavorable factors, including high-sugar diets, poor hygiene, and certain parental habits, can induce dysbiosis and contribute to both oral and systemic diseases [157,158].

Despite these advances, there remain substantial knowledge gaps. In particular, the long-term effects of the infant oral microbiome on physical growth, immune maturation, and neurodevelopment remain poorly understood. Furthermore, most available findings are cross-sectional, which limits the ability to establish causal relationships. This underscores the urgent need for longitudinal, multiomics, and interdisciplinary approaches that integrate microbiology, nutrition, immunology, and behavioral sciences.

Recognizing the oral microbiome as an important factor in children’s health offers the opportunity to design preventive strategies from the earliest stages of life. These strategies should focus not only on individual behaviors but also on the family and community environments where children develop, ensuring that oral health promotion is integrated into broader child health policies.

## Figures and Tables

**Table 1:** Impact of diet and early feeding on the development of the infant oral and gut microbiome: Evidence from observational and clinical studies.

Study Design	Characteristic Population	Nutrition or Other Variables	Microbial Diversity	Citation
Longitudinal observational study. 96 breast milk samples.	39 healthy mothers	The participant’s diet was not evaluated	Predominant bacterial groups: *Lactobacillus* spp., *Streptococcus* spp., and *Enterococcus* spp. Increase in bacteria throughout lactation: ↑ *Bifidobacterium* spp. and *Enterococcus* spp.	[50]
Longitudinal observational study. Oral samples from infants.	9 infants and their mothers	Breast milk, though diet was not evaluated	*Streptococcus mitis* was present in all infants and remained ubiquitous across ages. At 2 months, 6 species were found in ≥75% of infants. At 6 months, 7 more species were acquired. At 9 and 12 months: 4 additional species in ≥75% of infants. Most infant species were also prevalent in mothers except *Streptococcus peroris, Leptotrichia* Arg j44 uncultured, and *Porphyromonas* HF001.	[44]
Longitudinal observational study. Saliva samples from infants.	39 infants aged 2–20 months, breastfed for 10 months	Breast milk vs. total absence of breastfeeding. Other dietary aspects were not addressed.	At 2 months, non-breastfed infants: higher oral bacterial diversity, ↑ *Veillonella* spp., and Bacteroidota. Breastfed infants: lower oral diversity, ↑ *Streptococcus mitis*.	[58]
Cross-sectional observational study. Saliva samples from healthy neonates	Healthy neonates divided into two groups according to the type of feeding	Breast milk vs. exclusive infant formula	Both groups dominated by the phylum Bacillota. Formula-fed infants: **↑** Bacteroidota Breastfed infants: **↑** Actinomycetota and Pseudomonadota. *Streptococcus* spp. showed an increasing trend between weeks 4 and 8 in both groups.	[57]
Randomized Controlled Clinical Trial	394 healthy infants 4 to 6 months of age	Mediterranean Diet vs. industrial baby food	Greater abundance of beneficial taxa at 4 years. Increased microbial diversity at age 4	[59]
Longitudinal observational study. Saliva samples from newborns.	Babies from 0 to 180 days old	Breast milk	Early colonizers: *Streptococcus* spp. and *Staphylococcus* spp. Late colonizers: *Fusobacterium* spp., *Prevotella* spp., *Porphyromonas* spp., *Granulicatella* spp., and *Veillonella* spp.	[56]
Longitudinal cohort study. Tongue and cheek swabs.	12 infants, 0–6 months	Varied diet: breast milk, formula, and solid foods	Breast milk: lower oral bacterial diversity. **↑** *Streptococcus* spp. and ↓ *Prevotella* spp. Formula/solids: greater oral diversity. **↑** *Prevotella* spp. and Fusobacteriota.	[60]
Observational longitudinal study. Saliva samples from Swedish children at 6, 12, and 24 months of age.	59 healthy children	Breast milk and antibiotic use	First two years of life: Significant increase in alpha diversity and decrease in beta diversity Breast milk and antibiotic use were associated with relative abundance of specific OTUs. ↓ from the family *Streptococcaceae*.	[22]
Observational cross-sectional study. CC cohort: children with dental caries. CH cohort: healthy teeth in the same children. HH Cohort: Healthy children without cavities.	40 children from 3 to 6 years old	Participants’ diets were not assessed	Cohort CC: **↑** *Lactobacillus* spp., *Veillonella* spp. y *Prevotella.* Cohort CH: **↑** *Actinomyces* spp., *Bifidobacterium* spp. y *Abiotrophia* spp. Cohort HH: **↑** *Neisseria* spp., *Leptotrichia* spp., *Porphyromonas* spp., and *Gemella* spp.	[28]
Observational cross-sectional study. Oral and intestinal microbiota samples from children with and without obesity.	30 children with obesity and 30 children with normal weight, aged 3 to 5 years	Participants’ diets were not assessed	Children with obesity: Lower bacterial diversity. **↑** Bacillota and ↓ Bacteroidota. Normal-weight children: Increased microbial diversity	[61]
Observational longitudinal study. Saliva samples from children and their parents	27 healthy boys and 27 girls	Participants’ diets were not assessed	First week of life: **↑** *Streptococcus* spp., *Rothia* spp., and *Gemella* spp. Between 6 and 18 months: **↑** *Neisseria* spp., *Haemophilus* spp., and *Fusobacterium* spp. Between 36 and 60 months: no significant change compared to adults	[19]
Observational longitudinal study. Saliva samples from children and their mothers. Evaluation of caries in children.	134 healthy children, from 2 months to 4 years of age	Participants’ diets were not assessed	From 1.9 months to 39 months of age: **↑** alpha diversity from 31 OTUs to 84 OTUs. In individuals with caries: greater abundance of *Streptococcus mutans*.	[62]
Cross-sectional observational study. Saliva samples from children and their parents.	40 18-month-old children and their parents (mother and father)	Participants’ diets were not assessed	Microbial composition was significantly different between children and parents. Children’s microbiome was more similar to that of their mothers. In infants: **↑** Pseudomonadota and Fusobacteriota. In parents: **↑** Bacteroidota. Bacterial genera highly shared between infants and their parents: *Granulicatella* spp., *Streptococcus* spp., *Veillonella* spp., *Neisseria* spp., *Haemophilus* spp., *Rothia* spp. y *Fusobacterium* spp.	[63]
Cross-sectional observational study. Samples of gingival crevicular fluid, back of the tongue, and saliva	Healthy individuals of different age groups, from infancy to old age	No specific foods were evaluated	Decrease in alpha diversity and increase in beta diversity as age increases.	[64]
Observational cross-sectional study. Samples from different oral niches.	20 healthy adults	Participants’ diets were not assessed	Significant differences in alpha diversity between the different sampled sites (*p* < 0.0001), but not between individuals (*p* = 0.876).	[9]
Observational cross-sectional study. Questionnaire to assess lifestyle habits	39 schoolchildren aged 12 to 17 years old	Consumption of industrial pastries, sandwiches, and soft drinks	The oral microbiome was not analyzed directly. Significant associations were found between certain habits and oral health: frequency of tooth brushing (*p* = 0.005), consumption of pastries (*p* = 0.02), and consumption of soft drinks (*p* = 0.011).	[65]
Cross-sectional observational study. Saliva samples from adolescents	1500 teenage girls	Analysis of general dietary habits, hygiene, and water composition	Two main patterns, “stomatotypes”, were identified: one dominated by *Neisseria* spp. and *Haemophilus* spp., and the other by *Prevotella* spp. and *Veillonella* spp. Water composition was associated with variations in bacterial genera	[66]
Cross-sectional observational study. Salivary microbiota profiles were analyzed	Two families: including parents and their adult children	No specific foods were evaluated	Predominant bacterial phyla in both families: Bacillota, Bacteroidota, Pseudomonadota, Fusobacteriota, and Actinomycetota. Individuals from the same family have bacterial similarity, with greater similarity between mothers and adult children.	[67]
Cross-sectional and longitudinal observational study. Biofilm and saliva samples.	64 17-year-old adolescents with low caries prevalence and access to dental care from childhood	No specific foods were evaluated	In saliva: Bacillota was the predominant phylum (48%) and Actinomycetota (20%). In dental biofilm: Bacillota and Actinomycetota with similar abundance. In caries participants: ↑ *Scardovia wiggsiae, Streptococcus mutans*, *Bifidobacterium longum*, *Leptotrichia* spp. HOT498 and *Selenomonas* spp. in saliva. In dental biofilm, there was **↑** *Corynebacterium matruchotii*.	[68]
Longitudinal study. Saliva analysis at three points in time over 10 years	107 individuals, including twins, from 12 to 24 years of age	No specific foods were evaluated	Twins shared more similar bacterial communities with each other. This similarity decreased with age and separation from the shared household. It is suggested that the environment has a greater impact than genetics. The most abundant genera: *Veillonella* spp., *Actinomyces* spp., and *Streptococcus* spp.	[69]
Randomized controlled clinical trial. Gingival parameters were evaluated.	42 adults with gingivitis	Mediterranean Diet vs. Ultra-processed products	With a Mediterranean diet: significant reduction in the gingival index and bleeding on probing. Both groups: no change in plaque buildup	[70]
Longitudinal study of nutritional intervention. Analysis of saliva samples	29 elite male athletes	High-carb vs. high-carb diet. Periodized vs. Periodized Carbs Low in carbohydrates and high in fat	Low-carb, high-fat diet: ↓ *Neisseria* spp. and **↑** *Streptococcus* spp. No significant changes in alpha diversity in the three groups. For athletes, the low-carb, high-fat diet can alter the composition of the oral microbiome	[71]
Cross-sectional observational study. Samples of supragingival plate	40 adolescents with and without dental caries	Consumption of free sugars	Cavities and low sugar consumption: **↑** *Lactobacillus* spp., *Streptococcus mutans*, *Actinomyces gerencseriae*, *Actinomyces dentails, Candida albicans*, *Scardovia wiggsiae*, and *Parascardovia acidifaciens*. No cavities and high sugar consumption: more balanced microbiota, despite high sugar consumption.	[72]
In vitro *experimental study* using a human saliva model	Saliva samples from healthy adults were used to create biofilms of *Streptococcus mutans* and *Candida albicans*	Glucose, fructose, sucrose, starch, and starch-sucrose combinations	Sucrose and a combination of sucrose with starch: **↑** formation of bacterial-fungal aggregates in saliva. Dense biofilms were developed. Glucose and fructose: more dispersed biofilms with less acid production were developed.	[73]

**↑** The population increases, **↓** The population decreases.

**Table 2:** Evolution of the oral microbiome in different age groups.

Age Range of Children	Growth of Oral Microbial Diversity	Citation
Neonates 0–8 weeks of age	↑ *Streptococcus* spp. and phylum Bacillota.	[53]
0–2 months of age	↑ *Bifidobacterium* spp. and *Enterococcus* spp. *Streptococcus mitis* remained ubiquitous across ages.	[42]
4–6 months of age	Early colonizers *Streptococcus* spp. and *Staphylococcus* spp.	[74]
0–6 months of age	*Fusobacterium* spp., *Prevotella* spp., *Porphyromonas* spp., *Granulicatella* spp., and *Veillonella* spp.	[52]
6, 12, and 24 months of age.	↑ Firmicutes and Proteobacteria	[22]
1 year old	**↑** Pseudomonadota and Fusobacteriota.	[75]
3–5 years old	*Neisseria meningitidis*, *Streptococcus pneumoniae*, and *Haemophilus parainfluenzae*.	[76]
3–6 years old	**↑** *Neisseria* spp., *Leptotrichia* spp., *Porphyromonas* spp., and *Gemella* spp.	[28]
11–15 years old	**↑** *Streptococcus* spp., *Prevotella* spp., *Neisseria* spp., and *Rothia* spp.	[77]
12–24 years old	**↑** *Veillonella* spp., *Actinomyces* spp., and *Streptococcus* spp.	[78]

**↑** The population increases, **↓** The population decreases.

## Data Availability

No new data were created in this study.
